# Turning the Surface Electronic Effect Over Core‐Shell CoS_2_─Fe_x_Co_1‐x_S_2_ Nanooctahedra Toward Electrochemical Water Splitting in the Alkaline Medium

**DOI:** 10.1002/advs.202411622

**Published:** 2024-11-28

**Authors:** Lian‐Ming Lyu, Yu‐Chung Chang, Han‐Jung Li, Pei‐En Wang, Ruei‐Hung Juang, Ming‐Yen Lu, Cheng‐Shiuan Li, Chun‐Hong Kuo

**Affiliations:** ^1^ Department of Applied Chemistry National Yang‐Ming Chiao Tung University No. 1001, Daxue Rd. East Dist. Hsinchu 300093 Taiwan; ^2^ Department of Materials Science and Engineering National Tsing Hua University 101, Section 2, Kuang‐Fu Road Hsinchu 300044 Taiwan; ^3^ Green Energy and Environment Research Laboratories Industrial Technology Research Institute 195, Sec. 4, Chung Hsing Rd., Chutung Hsinchu 310410 Taiwan; ^4^ Department of Applied Chemistry and Center for Emergent Functional Matter Science National Yang‐Ming Chiao Tung University No. 1001, Daxue Rd. East Dist. Hsinchu 300093 Taiwan; ^5^ National Synchrotron Radiation Research Center 101 Hsin‐Ann Road, Hsinchu Science Park Hsinchu 300092 Taiwan

**Keywords:** cobalt, electronic effect, hydrogen evolution reaction, iron, oxygen evolution reaction

## Abstract

The long‐term challenge in overall water splitting is the conflict in the pH condition of electrolytes for achieving efficient hydrogen evolution reaction (HER) and oxygen evolution reaction (OER) at the same time, in addition to the typical cost issue in catalysts. It hence raises an intense research interest in seeking cost‐efficient non‐noble metal electrocatalysts as well as compromising electrolyte conditions for electrocatalytic HER and OER. To tackle the problems, various approaches are demonstrated to engineer the electronic effect on the active sites of catalysts for enhancing the activities. In this work, the core‐shell CoS_2_─Fe_x_Co_1‐x_S_2_ nanooctahedra is fabricated with a tunable Fe content over the surface and took them as the model catalyst for systematic studies in alkaline OER and HER. By various X‐ray spectroscopies as well as electron microscopy, the results showed that the shells of CoS_2_─Fe_x_Co_1‐x_S_2_ nanooctahedra formed the {111} surfaces of Fe_0.9_Co_1.0_S_2_ and Fe_0.25_Co_0.75_S_2_ with and without the promotion by OH^−^ anions during the syntheses. Catalyzed by the CoS_2_, Fe_0.25_Co_0.75_S_2_, and Fe_0.9_Co_1.0_S_2_ {111} surfaces, the results of alkaline OER and HER indicated the Fe_0.9_Co_1.0_S_2_ the most superior activities by virtue of the optimized Fe─Co electronic effect. From the predictions by density functional theory (DFT) calculations in reaction thermodynamics, the energy barriers in OER and HER both follow the order of Fe_0.9_Co_0.1_S_2_(111) < Fe_0.25_Co_0.75_S_2_(111) < CoS_2_(111). However, FeS_2_(111) is worse than Fe_0.9_Co_0.1_S_2_(111). From the confirmations by in‐situ X‐ray spectroscopies in reaction kinetics, the Co sites of Fe_0.9_Co_0.1_S_2_(111) on the core‐shell nanooctahedra exhibited much higher activities than those of CoS_2_(111) under the applied potentials for OER and HER, which reflected the electronic benefits from the existing Fe neighbors.

## Graphical abstract

This work fabricates core‐shell CoS_2_─Fe_x_Co_1‐x_S_2_ nanooctahedra, finding that Fe_0.9_Co_0.1_S_2_ {111} surfaces show superior oxygen evolution reaction (OER) and hydrogen evolution reaction (HER) activity in alkaline conditions due to optimized Fe─Co electronic effects.

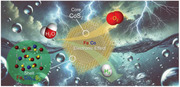

## 1 Introduction

Hydrogen gas presents a high‐potential energy alternative to fossil fuels, emitting almost zero carbon when it's used in fuel cell processes, where the only byproduct is H_2_O.^[^ ^1^, ^2^, ^3^ ^]^ Among various hydrogen sources, the green hydrogen gas produced through the hydrogen evolution reaction (HER) at the cathode in electrochemical water splitting has an exceptionally low carbon footprint.^[^ ^4^, ^5^, ^6^ ^]^ However, the kinetics of oxygen evolution reaction (OER) at the anode is usually sluggish, which involves a four‐electron transfer, and significantly hinders the energy efficiency of electrochemical water splitting.^[^ ^7^ ^]^ Some commercial catalysts for OER, such as IrO_2_ and RuO_2_, can have adequate activities in alkaline media. Unfortunately, they also face limitations arising from their high cost, scarcity, and instability. The conflict in the pH condition of electrolytes for efficient HER (acidic) and OER (basic) also sets a great challenge in achieving overall water splitting. For these reasons, there is an intense research interest in seeking cost‐efficient non‐noble metal electrocatalysts as well as compromising electrolyte conditions for both electrocatalytic HER and OER.

Pyrite‐type metal sulfides (such as FeS_2_, NiS_2_, and CoS_2_) have garnered significant attention due to their abundance, controllable morphologies, and tunable electronic properties.^[^ ^8^, ^9^, ^10^ ^]^ Among them, CoS_2_ received more attention as it has relatively better activity in water splitting than FeS_2_, and NiS_2_.^[^ ^11^, ^12^, ^13^, ^14^, ^15^ ^]^ In the structure of CoS_2_, the octahedral sites of Co^2+^ are split into t_2g_ and e_g_ sub‐bands by the crystalline field.^[^ ^16^ ^]^ A higher filling occupancy of the e_g_ band of transition metal ions has been linked to the enhanced water‐splitting activity in alkaline solutions.^[^ ^17^ ^]^ However, the intrinsic electrocatalytic performance of CoS_2_ materials is hindered by the low exposure of active sites and inferior reaction kinetics. To tackle the challenges, various approaches have been demonstrated, for example heterostructure fabrication (Co_3_O_4_/CoS_2_,^[^ ^15^ ^]^ CoS_2_@MoS_2_@NiS_2_,^[^ ^18^ ^]^ CoS_2_@MoS2,^[^ ^19^ ^]^ Cu@CoS_2_,^[^ ^20^ ^]^ and Pt‐CoS_2_^[^ ^21^ ^]^) and heteroatom doping (Se‐doped CoS_2_,^[^ ^22^ ^]^ N‐doped CoS_2_,^[^ ^14^ ^]^ W‐doped CoS_2_,^[^ ^23^ ^]^ and Fe─CoS_2_^[^ ^24^, ^25^, ^26^, ^27^, ^28^ ^]^), to engineer the electronic effect on the Co sites for enhancing water‐splitting performance. Nevertheless, a limited number of reports demonstrated systematic manipulations in the compositions and structures of specific surfaces by modulating the kinetic growth with a facile chemical approach. It hence lacks deep investigation into the correlation between the catalyst electronic effect and the OER/HER activity to provide more objective insights into both reaction thermodynamics and kinetics.

**Figure 1:**
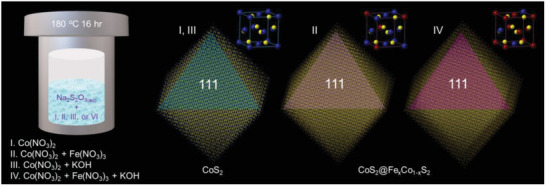
Brief interpretation for the synthetic conditions of CoS_2_ and core‐shell CoS_2_─Fe_x_Co_1‐x_S_2_ nanooctahedra.

In this work, we have successfully fabricated the core‐shell CoS_2_─Fe_x_Co_1‐x_S_2_ nanooctahedra with a tunable Fe content over the surface by a hydrothermal method (**Figure** **1**), and used them as the model catalyst for systematic studies in alkaline OER and HER. The core‐shell nanooctahedra, including their crystal, electronic, and surface structures, were carefully characterized and confirmed by synchrotron powder X‐ray diffraction (SPXRD), synchrotron X‐ray absorption (SXAS), synchrotron X‐ray emission (SXES), and high‐resolution X‐ray photoluminescence spectroscopies (HRXPS) in addition to typical analyses by electron microscopy (EM). The results showed that the shells of CoS_2_─Fe_x_Co_1‐x_S_2_ nanooctahedra formed the {111} surfaces of Fe_0.9_Co_1.0_S_2_ and Fe_0.25_Co_0.75_S_2_ with and without the promotion by OH^−^ anions during the syntheses. Catalyzed by the CoS_2_, Fe_0.25_Co_0.75_S_2_, and Fe_0.9_Co_1.0_S_2_ {111} surfaces, the results of alkaline OER and HER indicated the Fe_0.9_Co_1.0_S_2_ had the most superior activities by virtue of the optimized Fe─Co electronic effect. The activity enhancement by the electronic effect was finally validated and interpreted by density functional theory (DFT) calculations for reaction thermodynamics and in‐situ X‐ray spectroscopies for reaction kinetics.

## 2 Results and Discussion

### 2.1 CoS_2_ Nanooctahedra with Fe─Modified Surfaces

**Figure 2:**
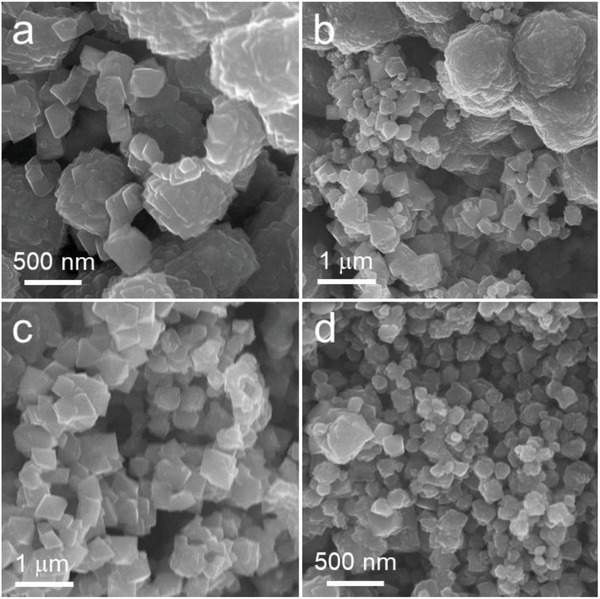
SEM images of the synthesized pyrite products labeled as a) CoS_2_, b) OH─CoS_2_, c) Fe─CoS_2_, and d) Fe─OH─CoS_2._

In our previous works, we discovered that the pyrite nanostructures (cubic) could be obtained instead of spinel ones by using Na_2_S_2_O_3_ to replace Na_2_S.^[^ ^29^, ^30^ ^]^ In addition, the Co‐based pyrite nanostructures (CoS_2_ and Ni_x_Co_1‐x_S_2_) formed in an octahedral shape with exposed {111} surfaces. According to the results of X‐ray absorption spectroscopy (XAS) and density function theory (DFT) calculations, we have realized the role of Ni‐Co electronic effect which was able to promote the activity and stability of pyrite Ni_x_Co_1‐x_S_2_ nanostructures in the electrochemical hydrogen evolution reaction (HER) in the aqueous alkaline medium, and the Co sites were the major active sites.^[^ ^30^ ^]^ Those observations thus interest us to look into the influence of Fe─Co electronic effect in alkaline water splitting based on rationally designed Fe_x_Co_1‐x_S_2_ surfaces over the pyrite nanostructures. For this aim, we demonstrate the hydrothermal method that successfully forms CoS_2_ and core‐shell CoS_2_─Fe_x_Co_1‐x_S_2_ nanooctahedra with tunable Fe contents on the shell {111} surfaces, as interpreted in Figure 1. Their corresponding unit cells are also displayed as the insets (Co: blue, S: yellow, and Fe: red). There are four different conditions employed for getting optimized core‐shell CoS_2_─Fe_x_Co_1‐x_S_2_ nanooctahedra. The products obtained in those conditions were labeled as CoS_2_ (I), Fe─CoS_2_ (II), OH─CoS_2_ (III), and Fe─OH─CoS_2_ (IV) according to the used reactants. The CoS_2_ (I) was obtained only with Co(NO_3_)_2_, the Fe─CoS_2_ (II) with Co(NO_3_)_2_ and Fe(NO_3_)_3_, the OH─CoS_2_ (III) with Co(NO_3_)_2_ and KOH, and the Fe─OH─CoS_2_ (IV) with Co(NO_3_)_2_, Fe(NO_3_)_3_ and KOH. All syntheses were surfactant‐free to avoid surface contamination.^[^ ^30^ ^]^ **Figure** **2** shows the scanning electron microscope (SEM) images of the synthesized products in conditions I‐IV. The results reveal that the particles of CoS_2_ and OH─CoS_2_ form nanooctahedra but most of them aggregated into hollow microspheres with 2.1 and 1.6 µm in diameters, respectively (Figure 2a,b; Figure S1a,b, Supporting Information). In contrast, the particles of Fe─CoS_2_ (II) and Fe─OH─CoS_2_ (IV) are mostly well‐dispersive nanooctahedra with sizes of 485.6 and 182.5 nm (Figure S1c,d, Supporting Information), indicating some possible changes in the particle surface property.

**Figure 3:**
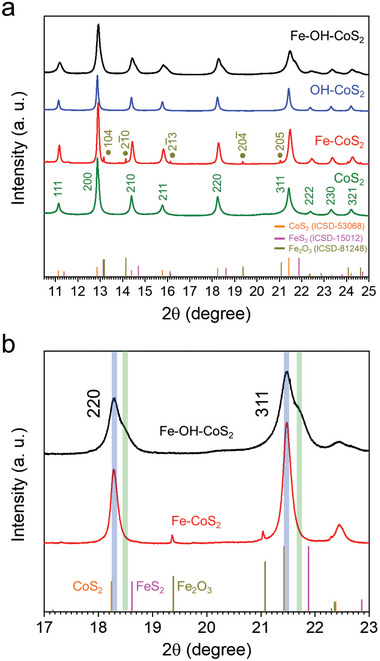
a) Synchrotron powder X‐ray diffractions patterns of the synthesized pyrite products, b) the corresponding patterns of Fe─OH─CoS_2_ and Fe─CoS_2_ shown in a selected range of 2‐theta degrees.

To analyze the crystal structures of the products, synchrotron powder X‐ray diffraction (SPXRD) spectroscopy was carried out using the X‐rays with 20 keV (λ = 0.61992 Å). **Figure** **3** collects the SPXRD patterns of CoS_2_, OH─CoS_2_, Fe─CoS_2_ and Fe─OH─CoS_2_. The peaks of cubic CoS_2_ (ICSD 53068), cubic FeS_2_ (ICSD 15012), and trigonal Fe_2_O_3_ (ICSD 81248) are also displayed for reference. In Figure 3a, the patterns of CoS_2_ (green) and OH─CoS_2_ (blue) reveal the pure CoS_2_ crystal structures without any peak from the byproduct. Hence, we believe that the role of OH^−^ was not kinetically critical in the formation process of CoS_2_ nanostructures. In the pattern of Fe─CoS_2_ (red), the major peaks fit with the phase of pyrite, but they are unsymmetrical and shift a bit to higher 2‐theta angles from those of pure CoS_2_. Meanwhile, weak byproduct peaks corresponding with those of Fe_2_O_3_ can be observed. The pattern of Fe─OH─CoS_2_ also shows unsymmetrical peaks fitting with the pyrite structure while the phase of Fe_2_O_3_ is no more observed. It's worth noting that the unsymmetrical peaks of Fe─OH─CoS_2_ all have obvious shoulders at higher 2‐theta angles. Figure 3b shows the 2‐theta range for 220 and 311 peaks of Fe─CoS_2_ and Fe─OH─CoS_2_. The 2 peaks of them obviously located in between those of CoS_2_ and FeS_2_ (blue marks). In addition, the significant shoulders at the 220 and 311 peaks of Fe─OH─CoS_2_ are also located in between those of CoS_2_ and FeS_2_ but stay closer to FeS_2_. For estimating the quantities of Fe bedded in the CoS_2_ structures, Rietveld refinement was performed on the four SPXRD patterns (Figure S2, Supporting Information). Table S1 (Supporting Information) summarizes the quantitative ratios, and the results of Rietveld refinement for all samples are recorded in Tables S2–S5 (Supporting Information). Specifically, we realized that the unsymmetrical peaks and shoulders in the XRD patterns of Fe─CoS_2_ and Fe─OH─CoS_2_ resulted from the formation of Fe_x_Co_1‐x_S_2_ shells. According to the results of Tables S1–S5 (Supporting Information), the components in the Fe─CoS_2_ contain CoS_2_, Fe_0.248_Co_0.752_S_2_, and Fe_2_O_3_ in a phase ratio of 63.2%, 34.3%, and 2.5% (Tables S1 and S4, Supporting Information). Those in the Fe─OH─CoS_2_ contain CoS_2_ and Fe_0.904_Co_0.096_S_2_ in a phase ratio of 78.9% and 21.1%. (Tables S1 and S5, Supporting Information) The separated phases infer the heterostructures in the nanooctahedra, and the high percent phase of CoS_2_ reflects the possible dense core of CoS_2_ in a core‐shell nanocrystal. Table S6 (Supporting Information) records that the high percent elemental ratio of Co to Fe is about 91.45% to 8.55% in the Fe─OH─CoS_2_, suggesting the possible existence of dense CoS_2_ cores as well. To validate our assumption, bright‐field transmission electron microscope (BF‐TEM) imaging and high‐angle annular dark filed‐scanning transmission electron microscope‐energy dispersive X‐ray (HAADF‐STEM‐EDX) analyses were utilized to look for the evidence.

**Figure 4:**
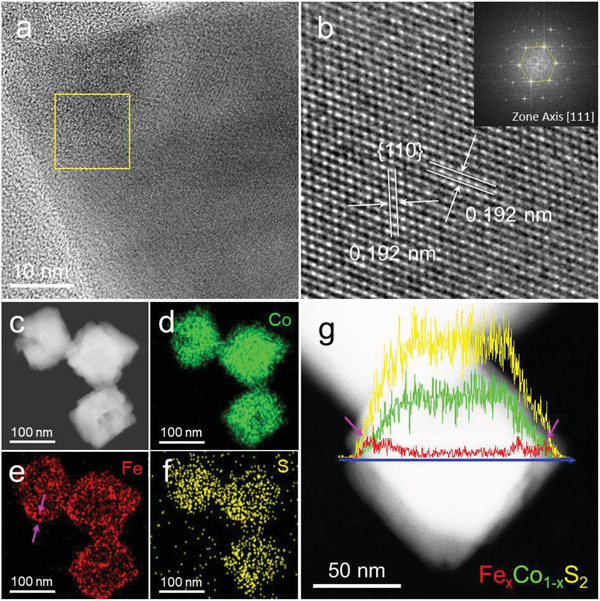
a) The BF‐TEM image at one corner of the nanooctahedron from the Fe─OH─CoS_2_ sample. b) The high‐resolution BF‐TEM image acquired from the marked area in (a). The inset panel is the corresponding FFT pattern over the same area. c) The HAADF‐STEM image of the nanooctahedra from the Fe─OH─CoS_2_ sample, and their corresponding EDX maps of d) Co, e) Fe, and f) S. The HAADF‐STEM image of a single nanooctahedron as in (a), and its EDX line‐scan profile.

**Figure** **4**a is the BF TEM image at one corner of the nanooctahedron from the Fe─OH─CoS_2_ sample. Figure 4b shows the high‐resolution TEM (HRTEM) image over the yellow‐square area where the 2D arrangements of lattice fringes could be resolved. The values of lattice d‐spacing along two different directions are both 0.192 nm, in‐between the values of CoS_2_ (220) and FeS_2_ (220) crystal planes (1.908 and 1.953 nm), and thus fit with the {110} crystal planes. The inset panel is the fast Fourier transformation (FFT) pattern from the 2D arrangements of lattice fringes in Figure 4b. The hexagonal pattern of spots indicates the [111] direction viewing on the projection face as well as the {111} crystal face on the surface of a nanooctahedron. Figure 4c is the HAADF‐STEM image of multiple nanooctahedra from the Fe─OH─CoS_2_ sample, and their corresponding EDX maps of Co, Fe, and S are displayed in Figure 4d–f and Figure S11e (Supporting Information). Notably, the distributions of Co and S are homogeneous over the particles while that of Fe is more concentrated along the contours (pink arrows in Figure 4e). It can be further confirmed by the line‐scan profile across the diagonal corners of a single nanooctahedron which clearly shows significantly higher abundances at the two edges (pink arrows in Figure 4g). The results are great evidence to support the proposed model of core‐shell CoS_2_─Fe_x_Co_1‐x_S_2_ nanooctahedra. In comparison, Figure S3 (Supporting Information) collects the HAADF‐STEM images of CoS_2_, OH─CoS_2_, and Fe─CoS_2_ along with their corresponding results of STEM‐EDX mapping. The distributions of Co and S are homogeneous for all samples, and that of Fe is more concentrated along the contours of particles (the pink arrow). All the results support the observations from SPXRD and indicate the capability of modulating particle compositions based on our synthetic conditions.

After ensuring the conditions for forming CoS_2_ and core‐shell CoS_2_─Fe_x_Co_1‐x_S_2_ nanooctahedra, a control experiment by only adding Fe^3+^ without Co^2+^ in the synthesis was further performed. The goal of this condition, Fe─OH, was trying to make pure FeS_2_ nanooctahedra. Unfortunately, only Fe_2_O_3_ nanoplates were able to be obtained. Figure S4a (Supporting Information) presents the PXRD pattern of the Fe─OH sample. All peaks correspond with those of the trigonal Fe_2_O_3_ crystal structure (ICSD 81 248). Figure S4b (Supporting Information) is the SEM image showing the Fe─OH sample comprising Fe_2_O_3_ nanoplates. Figure 4c,d are the BF‐TEM image of a single nanoplate and its corresponding selected‐area electron diffraction (SAED) pattern over the yellow‐marked area. The results show that the large flat face on the single nanoplate should be (0001).

### 2.2 Electronic Structures of CoS_2_ and CoS_2_─Fe_x_Co_1‐x_S_2_ Nanooctahedra

The oxidation states of different elements on the surfaces of nanooctahedra have been analyzed using high‐resolution X‐ray photoelectron spectroscopy (HRXPS). In Figure S5a–d (Supporting Information), the deconvoluted Co 2p spectra show that all CoS_2_ and Fe_x_Co_1‐x_CoS_2_ surfaces comprise Co^3+^ (red) and Co^2+^ (dark green). Specifically, the Co^3+^(2p_3/2_ and p_1/2_) peaks are at 779.0 and 794.0 eV while Co^2+^(2p_3/2_ and p_1/2_) at 780.7 and 795.7 eV. The grey areas are Co(2p) satellite peaks at 784.7 and 799.7 eV.^[^ ^31^, ^32^, ^33^ ^]^ All kinds of Co(2p) peaks don't have significant shifts in their positions in comparison among the samples, suggesting the stable Co valence state throughout the surface‐modification process. Figure S5e,f (Supporting Information) represents the deconvoluted Fe(2p) spectra coupled with a broad Auger peak of Co‐LMM.^[^ ^34^ ^]^ It is worth noting that only the peaks of Fe^2+^ (purple, 707.3, and 720.1 eV) are observed in the spectrum of Fe─OH─CoS_2_ beside the Fe(2p) satellite peaks.^[^ ^35^ ^]^ However, those of Fe^2+^ (purple, 710.2, and 723.3 eV) and Fe^3+^ (red, 712.4, and 725.1 eV) show up in the spectrum of Fe─CoS_2_.^[^ ^36^ ^]^ The existence of Fe^3+^ surely comes from the Fe_2_O_3_ in the majority because it is not observed in the Fe─OH─CoS_2_. The results hence release a critical clue that the role of OH^−^ must have something to do with the reduction of Fe^3+^ to Fe^2+^ toward the formation of Fe_x_Co_1‐x_CoS_2_. In Figure S6a–d (Supporting Information), the spectra of O(1s) show the peaks at around 532.5 and 531.8 eV which can be assigned to the adsorbed oxygen (green), and adsorbed oxygen in hydroxide and oxyhydroxide (orange), respectively. In the spectra of Fe─CoS_2_, an additional peak at 529.8 eV can be assigned to the lattice oxygen contributed by Fe_2_O_3_ (pink).^[^ ^37^, ^38^, ^39^ ^]^ The result well corresponds with the spectrum of Fe(2p) in Figure S5e (Supporting Information). On the other hand, the relative abundance of O_OH_ in the spectra of OH─CoS_2_ and Fe─OH─CoS_2_ are higher than those of the other two samples. The O_OH_ is particularly the highest in the Fe─OH─CoS_2_ sample_._ This phenomenon should have arisen from the addition of KOH that promotes hydroxylation over the nanooctahedral surfaces. Besides, the relative abundance of O_OH_ for the Fe─CoS_2_ is also higher than the CoS_2_. To our knowledge, the dissociation of H_2_O molecules leads to hydroxylation over the catalyst surfaces.^[^ ^38^ ^]^ It reflects that the Fe_x_Co_1‐x_S_2_ surfaces have higher H_2_O affinity toward hydroxylation. For this reason, we predict that H_2_O may be more reactive to the surfaces of core‐shell CoS_2_─Fe_x_Co_1‐x_S_2_ nanooctahedra in the alkaline water splitting. Figure S6e–h (Supporting Information) shows the deconvoluted HRXPS spectra of S(2p) with the characteristic peaks in purple, yellow, and grey that can be assigned to the dimer S_2_^2−^ bonds in the lattices of pyrite structures, adsorbed polysulfide S_n_^2−^, and adsorbed SO_4_^2−^, respectively.^[^ ^40^ ^]^ The S(2p_3/2_) peaks of S_2_^2−^ in the spectra of OH─CoS_2_ and Fe─OH─CoS_2_ both are located at 163.3 eV. The value is a bit higher than those of CoS_2_ (163.0 eV) and Fe─CoS_2_ (163.1 eV), inferring the slight influence of O_OH_ abundances.^[^ ^41^ ^]^ In contrast with the lattice S_2_^2−^, the S(2p) peaks of the adsorbed SO_4_^2−^ are at the high binding energy in all spectra.^[^ ^42^ ^]^ As for the adsorbed polysulfide S_n_^2−^, we believe that it is the byproduct resulting from the complex chain reactions based on S_2_O_3_^2−^ and its derivatives. The existence of SO_4_^2−^ and S_n_^2−^ indicates that S_2_O_3_^2−^ actually underwent both oxidation and reduction reactions, revealing the clues for the formation mechanism of Fe_x_Co_1‐x_S_2_.

### 2.3 Speculated Formation Mechanism of Fe_x_Co_1‐x_S_2_ Crystal Structure

Given that the formation of Fe_x_Co_1‐x_S_2_ shells in a high Fe content requires KOH, the real function of OH^−^ is very likely to promote the production of Fe^2+^ from Fe^3+^ for Fe_x_Co_1‐x_S_2_ shells. We hence propose the possible formation mechanism of CoS_2_ and Fe_x_Co_1‐x_S_2_ in chemical reactions as below.

(1) $$8Fe^{3+}+S_{2}{O_{3}}^{2-}+10OH^{-}\to 8Fe^{2+}+2S{O_{4}}^{2-}+5H_{2}O$$

(2) $$2Fe^{3+}+6OH^{-}\to Fe_{2}O_{3}+3H_{2}O$$

(3) $$2S_{2}{O_{3}}^{2-}\to 2{S_{2}}^{2-}+3O_{2}$$

(4) $$M^{2+}\left({\mathrm{Co}}^{2+},{\mathrm{Fe}}^{2+}\right)+{S_{2}}^{2-}\to {\mathrm{MS}}_{2}\left(\mathrm{Co}S_{2},{\mathrm{Fe}}_{x}{\mathrm{Co}}_{1-x}S_{2}\right)$$

In general, the formation of Fe_2_O_3_ in Equation (2) is a major reaction in the alkaline medium under heating. However, the oxidation of S_2_O_3_^2−^ to form SO_4_^2−^ and release electrons in the alkaline medium as Equation (1) has been proven an easy and fast pathway.^[^ ^43^ ^]^ The XPS signals of SO_4_^2−^ were also observed over the surfaces of all synthesized samples (Figure S5e–h, Supporting Information). Therefore, we suggest that abundant Fe^2+^ cations would be largely produced with the addition of KOH, and hence result in Fe_x_Co_1‐x_S_2_ within a high Fe content (e.g., Fe─OH─CoS_2_). Otherwise, the competitive reaction for consuming Fe^3+^ by Equation (2) would be significant and lead to a certain amount of Fe_2_O_3_ (e.g., Fe─CoS_2_). On the other hand, the Co^2+^ from Co(NO_3_)_2_ can react with S_2_^2−^ to CoS_2_ quickly, and therefore the formation of CoS_2_ seeds. However, the Fe^3+^ from Fe(NO_3_)_3_ has to be reduced to Fe^2+^ ions before reacting with S_2_^2−^. It delays the formation of Fe_x_Co_1‐x_S_2_ crystal and makes the Fe_x_Co_1‐x_S_2_ overgrow on the CoS_2_ seed to form the core‐shell structure. In addition, it also induces the gradient distribution of Fe from the particle inside to the surface where the Fe content is abundant, as proved in Figure 4e,g.

In addition to Equation (2), there are possible side reactions for S_2_O_3_^2−^ as Equations (5) to (6) in the alkaline medium.

(5) $$S_{2}{O_{3}}^{2-}+2OH^{-}\to 2S^{2-}+2O_{2}+H_{2}O$$

(6) $$2S_{2}{O_{3}}^{2-}+4S^{2-}+6H_{2}O\to S_{8}+12OH^{-}$$

In the side reactions, S_2_O_3_^2−^ would be reduced to form S^2−^ and S_8_. In fact, neither S^2−^ nor S_8_ was found over the sample surfaces in the XPS spectra. Although the polysulfide S_n_^2−^ was observed, there was no spinel FeCo_2_S_4_ nanocrystal which would be obtained by S^2−^ as in our former results.^[^ ^30^ ^]^ The observations fully reveal Equations (5) a very minor reaction pathway in our syntheses. The role of OH^−^ is therefore an important switch to boost the general Fe^2+^ in the growth process.

### 2.4 Alkaline Water Splitting Catalyzed by CoS_2_─Fe_x_Co_1‐x_S_2_ Nanooctahedra

Efficient water splitting, including oxygen and hydrogen evolution reactions (OER and HER), catalyzed by low‐cost catalysts instead of Pt has been a long‐term target for scientists. One of the major challenges is to look for the electrocatalysts that can drive OER and HER in high efficiency together in the same electrolyte condition. In this regard, we specially examined the performances of alkaline OER and HER catalyzed by the core‐shell CoS_2_─Fe_x_Co_1‐x_S_2_ nanooctahedra. In brief, all the synthesized products were used as the electrocatalysts loaded on the carbon papers to form the working electrodes. The electrodes were determined for their electrochemically active surface areas (ECSA) prior to the experiments for alkaline water splitting. ECSAs for electrocatalysts could be estimated from the electrochemical double‐layer capacitance (C_dl_) over the catalytic surfaces. The relationship between the double‐layer charging current *i_c_* and C_dl_ is as the Equation (7), where *v* denotes the scan rate in cyclic voltammograms (CV).^[^ ^44^ ^]^

(7) $$i_{c}=vC_{\mathrm{dl}}$$

Figure S7a–e (Supporting Information) collects the CVs at the OCP for CoS_2_, OH─ CoS_2_, Fe─CoS_2_, Fe─OH─CoS_2_, and Fe_2_O_3_ at different scan rates (*r*) of 10, 20, 40, 60, 80, and 100 mV s^−1^. From their corresponding plots of charging current (∆*I*/2) versus scan rate (Figure S7f, Supporting Information), where ∆*I* = *I*_top_ − *I*_bottom_ at OCP, the obtained slopes represent the values of C_dl_ for the electrocatalysts which are 2.48 mF for CoS_2_, 2.50 mF for OH─CoS_2_ 2.11 mF for Fe─CoS_2_, 5.60 mF for Fe─OH─CoS_2_, and 0.24 mF for Fe_2_O_3_. The values of their ECSA hence are 62.00, 62.50, 52.75, 140, and 6.00 cm^−2^ for CoS_2_, OH─CoS_2_, Fe─CoS_2_, Fe─OH─CoS_2_, and Fe_2_O_3_, determined by C_dl_/C_s_ (see the experimental section).

**Figure 5:**
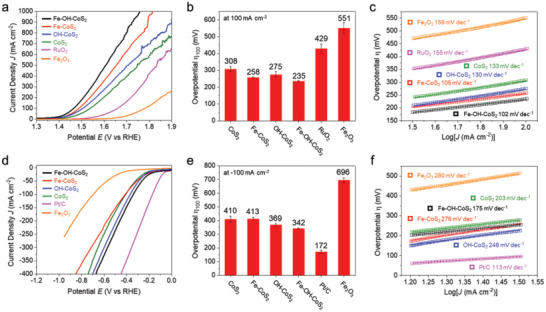
The results of electrocatalytic a–c) OER and d–f) HER measured in 1 M KOH electrolyte. (a, d) Polarization curves for CoS_2_, OH─CoS_2_, Fe─CoS_2_, Fe─OH─CoS_2_, Fe_2_O_3_, RuO_2_ (OER), and Pt/C (HER) at 50 mV/s. (b, e) Overpotentials required for current density at 100 mA/cm^2^ (OER), and −100 mA/cm^2^ (HER). (c, f) Tafel plots obtained from the polarization curves.

**Figure** **5** collects the results of OER (Figure 5a–c; Figure S8, Supporting Information) and HER (Figure 5d–f) catalyzed by our home‐made working electrodes in the aqueous electrolyte of 1 M KOH. Form the LSV curves for OER (Figure 5a), the overpotentials (Figure 5b) were determined at the current density of 100 mA cm^−2^ (η_100_) and gave the order of Fe─OH─CoS_2_ < Fe─CoS_2_ < OH─CoS_2_ < CoS_2_ < RuO_2_ < Fe_2_O_3_. The values were 308, 275, 258, 235, and 551 mV (vs RHE) for CoS_2_, OH─CoS_2_, Fe─CoS_2_, Fe─OH─CoS_2_, and Fe_2_O_3_, respectively. The commercial RuO_2_ was also tested for reference whose overpotential was 429 mV. On the other hand, their Tafel slopes (Figure 5c) analyzed from Figure 5a were also in the same order of Fe─OH─CoS_2_ (102 mV dec^−1^) < Fe─CoS_2_ (105 mV dec^−1^) < OH─CoS_2_ (130 mV dec^−1^) < CoS_2_ (133 mV dec^−1^) < RuO_2_ (155 mV dec^−1^) < Fe_2_O_3_ (159 mV dec^−1^). The Tafel slope means how fast the current density increases against overpotential, which reflects the kinetics of catalyzed OER. The results reveal that the efficiency in the OER electron‐transfer process was gradually optimized upon the formation of Fe─rich Fe_x_Co_1‐x_S_2_ surfaces. It also infers the benefit from the Fe─Co electron effect toward alkaline OER. In contrast, the results in HER show the overpotentials at −100 mA cm^−2^ (Figure 5e) in the order of Pt/C (172 mV) < Fe─OH─CoS_2_ (342 mV) < OH─CoS_2_ (369 mV) < CoS_2_ (410 mV) < Fe─CoS_2_ (413 mV) < Fe_2_O_3_ (696 mV). The higher values than those of OER indicate the pyrite nanooctahedra the poor HER electrocatalysts in the alkaline medium. However, except for the Pt/C, the Fe─OH─CoS_2_ still had significantly lower HER overpotential than the others. For their Tafel slopes (Figure 5f), the order followed Pt/C (113 mV dec^−1^) < Fe─OH─CoS_2_ (175 mV dec^−1^) < CoS_2_ (203 mV dec^−1^) < OH─CoS_2_ (248 mV dec^−1^) < Fe─CoS_2_ (276 mV dec^−1^) < Fe_2_O_3_ (280 mV dec^−1^). The Fe─OH─CoS_2_ exhibited the best HER kinetics except for the commercial Pt/C, again suggesting the possible benefit from the Fe─Co electronic effect in the condition of high Fe abundance.

Figure S9 (Supporting Information) collects the Nyquist plots of the Fe─OH─CoS_2_, Fe─CoS_2_, OH─CoS_2_, and CoS_2_ catalysts by applying 1.45 V versus RHE (for OER Figure S9a, Supporting Information) and −0.45 V versus RHE (for HER Figure S9b, Supporting Information). In Figure S9a (Supporting Information), the plots of all catalysts at 1.45 V have two semicircles that correspond with a simple Randle's circuit (the inset scheme), where R_s_ denotes the internal resistance of the electrodes and electrolyte, R_ct_ means the charge‐transfer resistance at the interface between the working electrode and the electrolyte, R_f_ indicates the resistance of the deposited layers on the working electrode, CPE_1_ is the constant phase element of the deposited layer, and CPE_2_ is the double‐layer capacitance.^[^ ^45^, ^46^, ^47^, ^48^ ^]^ The two semicircles arise from the formation of oxide or oxyhydroxide surface layers over the pyrite nanooctahedra that exhibit distinct resistances apart from the sulfide catalysts.^[^ ^49^ ^]^ Table S7 (Supporting Information) summarizes the values of all terms in the equivalent circuit. Specifically, the values of R_ct_ are 5.05 Ω for Fe─ΟΗ‐CoS_2_, 9.34 Ω for OH─CoS_2_, 7.70 Ω for Fe─CoS_2_, and 12.06 Ω for CoS_2_, suggesting the best OER kinetics by the Fe─ΟΗ─CoS_2_. In contrast, the single‐semicircle plots were obtained for all catalysts at −0.45 V and could be fitted with an equivalent circuit composed of R_s_, R_ct,_ and CPE (the constant phase element of the deposited layer). These results correspond with those of alkaline HER in other reports.^[^ ^50^, ^51^ ^]^ The possible reason for this difference to Figure S9a (Supporting Information) is because the oxide or oxyhydroxide surface layers could not form easily under reduction at the negative potentials. Table S8 (Supporting Information) collects the values of all terms in the equivalent circuit in Figure S9b (Supporting Information). The values of R_ct_ are 14.00 Ω for Fe─ΟΗ─CoS_2_, 14.82 Ω for OH─ CoS_2_, 18.77 Ω for Fe─CoS_2_, and 28.77 Ω for CoS_2_. The Fe─ΟΗ─CoS_2_ is still the most efficient HER catalyst among the four.

To examine if there was a positive correlation between the Fe amount and Fe─Co electronic effect, we carried out the control experiments in which different amounts of Fe(NO_3_)_3_ were added to the syntheses to compare with the original Fe─OH─CoS_2_. They were labeled as 0.5Fe─OH─CoS_2_ and 1.5Fe─OH─CoS_2_ denoting a half and 1.5 times the original Fe(NO_3_)_3_ quantity. As shown in Table S9 (Supporting Information), the atomic ratios of Fe were 3.6% for 0.5Fe─OH─CoS_2_, 4.0% for 1.0Fe─OH─CoS_2_, and 9.8% for 1.5Fe─OH─CoS_2_. Figure S10 (Supporting Information) collects their corresponding OER and HER results in the electrolyte of 1 M KOH. From the LSV curves (Figure S10a,d, Supporting Information), the Tafel slopes for OER and HER were in the same order of 1.0Fe─OH─CoS_2_ < 0.5Fe─OH─CoS_2_ < 1.5Fe─OH─CoS_2_ (Figure S10c,f, Supporting Information). On the other hand, the overpotentials of 1.0Fe─OH─CoS_2_ in OER (at 100 mA cm^−2^) and HER (at −100 mA cm^−2^) were 235 and 342 mV, both the lowest values (Figure S10b,e, Supporting Information). However, the 1.5Fe─OH─CoS_2_ had a higher overpotential than that of the 0.5Fe─OH─CoS_2_ (403 vs 348 mV) in OER while the order reversed in HER (380 vs 419 mV). The activity decay over the 1.5Fe─OH─CoS_2_ was not as expected and thus further characterizations on these samples were performed to figure it out. Figure S11 (Supporting Information) displays the HAADF‐STEM‐EDX maps of 0.5Fe─OH─CoS_2_, 1.0Fe─OH─CoS_2_, and 1.5Fe─OH─CoS_2_. The overlaid image (Figure S11e, Supporting Information) and EDX maps of 1.0Fe─OH─CoS_2_ (Figure S11f–h, Supporting Information) are shown as the results in Figure 4. For the 0.5Fe─OH─CoS_2_, the distributions of Co and S are homogenously overlaid (Figure S11a, Supporting Information). That of Fe looks like surrounding outside CoS_2_ but does not form intact shells (Figure S11b–d, Supporting Information). Nevertheless, there was no significant segregation observed. For the 1.5Fe─OH─CoS_2_, the distributions of Co and S are also homogenously overlaid (Figure S11k,l, Supporting Information). However, phase segregation of Fe is clearly observed (Figure S11i–k, Supporting Information). We assume this phenomenon was induced by the boosted side reaction of Equation (2) due to the increased [Fe^3+^] in the synthesis, leading to forming much more Fe_2_O_3_ instead of Fe^2+^ by Equation (1). Therefore, it creates a limit for the Fe_x_Co_1‐x_CoS_2_ formation by simply increasing the amount of Fe^3+^ source in the synthesis. It also provides us a good understanding of how to optimize the Fe_x_Co_1‐x_CoS_2_ surface upon Fe^2+^ substituting for Co^2+^ over the nanooctahedral surfaces.

Given the superior activities of 1.0Fe─OH─CoS_2_ in OER and HER, the chronopotentiometry (CP) test for 100 h was finally carried out to confirm its durability (Figure S12, Supporting Information). In the curve for OER (red), the potential was slightly elevated from 1.58 to 1.78 V versus RHE after 100 h. However, the change was caused by not only the evolution of the catalyst but also the decrease in the [OH^−^].^[^ ^52^ ^]^ To exclude and clarify the effect of [OH^−^], the electrolyte of KOH was renewed at the 35th, 55th, and 80th hours. After every electrolyte renewal, the potential was lowered (1.67, 1.69, and 1.71 V) and gradually elevated, indicating the significant influence of [OH^−^] decrease. On the other hand, the curve for HER (black) is relatively smooth and stable from −0.38 to −0.44 V after 100 h without renewing the electrolyte because the mechanism of alkaline HER doesn't induce a significant [OH^−^] decrease.^[^ ^50^ ^]^

Figure S13 (Supporting Information) collects the HAADF‐STEM images and the elemental maps obtained by STEM‐EDX for the Fe─OH─CoS_2_ after OER and HER durability tests for 100 h. The results reveal that high oxygen abundances (Figure S13e,j, Supporting Information) were exhibited with a significant decrease in sulfur abundances (Figure S13d,i, Supporting Information) during OER and HER. Specifically, the final product from OER keeps the homogeneous distributions of Co, Fe, S, and O except for the low sulfur abundance and the high oxygen content in the structure. On the other hand, the product from HER has nanosilks as the byproduct (Figure S13f, Supporting Information). According to the maps of S and O (Figure S13i,j, Supporting Information), these nano silks are not metal sulfides but most metal oxides, hydroxides, or oxyhydroxides. However, a portion of metal sulfides may still exist in the product. Figure S14 (Supporting Information) collects the HRXPS spectra of Co(2p), Fe(2p), S(2p), and O(1s) for the Fe─OH─CoS2 after OER and HER durability tests for 100 h. After OER, the spectra of Co(2p) and Fe(2p) show that the valences of Co and Fe are both blended with 2+ and 3+ (Figure S14a,b, Supporting Information). It's worth noting that the spectrum of S(2p) only shows the peaks at 168.2 and 169.3 eV contributed by the sulfite groups of Nafion left on the electrode (Figure S14c, Supporting Information).^[^ ^53^ ^]^ Meanwhile, the spectrum of O(1s) has the peaks assigned to the adsorbed oxygen (green), and adsorbed oxygen in hydroxide and oxyhydroxide (orange), the lattice oxygen (pink), and the oxygen in the structure of Nafion (purple). In Figure S14e–h (Supporting Information), all the XPS spectra of the Fe─OH─CoS_2_ after HER show the same results as those after OER except that the little peaks of S_2_^2−^ from the residual pyrite sulfides, as proved by the Fe─shell in Figure S13h (Supporting Information) (the arrow). Given the results, we have got two clues that 1) all sulfur elements were leached under long‐term OER which made the formation of Co‐ and Fe─based oxides, hydroxides, or oxyhydroxides, 2) a portion of core‐shell CoS_2_─Fe_x_Co_1‐x_S_2_ nanooctahedra could exist under long‐term HER, and the byproduct of nano silks should be generated by the further oxidation or hydroxylation of the segregated Co^0^ and Fe^0^ over the nanooctahedron surfaces under negative potentials.^[^ ^30^ ^]^

We also examined the chronoamperometric (CA) results of CoS_2_, OH─CoS_2_, and Fe─CoS_2_ in 17 h as shown in Figure S15 (Supporting Information). In the short‐term tests, the CoS_2_ was quite stable in both OER and HER. The OH─CoS_2_, although not as stable as the CoS_2_, eventually reached stable after taking several hours in both OER and HER. For the Fe─CoS_2_, two drops occurred in both OER and HER (the arrows), if otherwise, the Fe─CoS_2_ exhibited good stability, too. The sudden drops were very likely from the evolution of adsorbed Fe_2_O_3_ under applied potentials or a bit of loss of catalysts.

### 2.5 Confirmation of Reaction Thermodynamics by DFT Calculations

**Figure 6:**
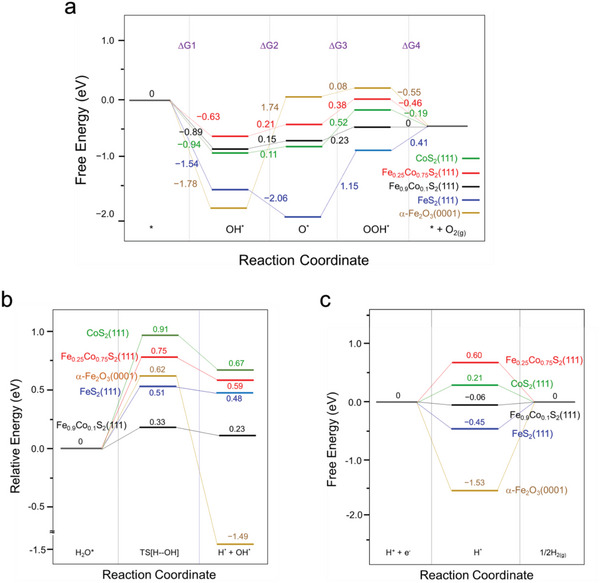
a) Free energy diagram of OER (ΔG) on various surfaces (U = 0.401 V, pH = 14). b) Energy diagram for breaking the H─OH Bond in the Volmer step on various surfaces. c) Free energy diagram of HER (ΔG_H*_) on various surfaces.

Density functional theory (DFT) calculations were conducted to elucidate the variations in alkaline OER and HER performances among specific surface models, including CoS_2_(111), Fe_0.25_Co_0.75_S_2_(111), Fe_0.9_Co_0.1_S_2_(111), FeS_2_(111), and α─Fe_2_O_3_(0001) as depicted in Figure S16 (Supporting Information). First of all, the OER free energy profiles for catalytic sites on specific surfaces, and their corresponding optimized structures of species involved in the OER (*OH, *O, and *OOH) on these surfaces are presented in **Figure** **6**a and Figure S17 (Supporting Information), respectively. In Figure 6a, the potential‐determining steps (PDS) for the OER over the pyrite surfaces of Co_2_(111), Fe_0.25_Co_0.75_S_2_(111), Fe_0.9_Co_0.1_S_2_(111), and FeS_2_(111) all occur at the third step (O* + OH⁻ → OOH* + e⁻, ΔG_3_) while that of α─Fe_2_O_3_(0001) at the second step (OH* → O* + H^+^ + e^−^, ΔG_2_). The free energy of PDS energy barriers are 0.23, 0.38, 0.52, and 1.15 eV for Fe_0.9_Co_0.1_S_2_(111), Fe_0.25_Co_0.75_S_2_(111), CoS₂(111), FeS₂(111) (ΔG_3_), and 1.74 eV for α─Fe_2_O_3_(0001) (ΔG_2_). It suggests that the OER activity follows the order of Fe_0.9_Co_0.1_S_2_(111) > Fe_0.25_Co_0.75_S_2_(111) > CoS_2_(111) > FeS_2_(111) > α─Fe_2_O_3_(0001). These computational results are consistent with those from the experimental observations showing the overpotentials follow the order: Fe─OH─CoS₂ < Fe─CoS₂ < CoS₂ < Fe₂O₃ (Figure 5b).

In our previous work, the alkaline HER activities by CoS_2_, NiS_2_, and NiCoS_2_ were evaluated by calculating both the barrier for water dissociation and the free energy of hydrogen adsorption using density functional theory (DFT).^[^ ^30^ ^]^ In the same way, the energy diagrams for H─OH bond dissociation in the Volmer step over the specific surfaces are examined and presented in Figure 6b, along with their corresponding structures of the initial, transition, and final states shown in Figure S18 (Supporting Information). The calculated energy barriers for H─OH bond dissociation are 0.33 eV for Fe_0.9_Co_0.1_S_2_(111), 0.51 eV for FeS_2_(111), 0.62 eV for α─Fe_2_O_3_(0001), 0.75 eV for Fe_0.25_Co_0.75_S_2_(111) and 0.91 eV for CoS_2_(111). These results indicate that the Fe_0.9_Co_0.1_S_2_(111) surface exhibits the highest HER activity in the Volmer step. The most stable adsorption sites for H on the specific surfaces are illustrated in Figure S19 (Supporting Information), and their corresponding free energy of H adsorption (ΔG_H*_) is presented in Figure 6c. The results indicate that the hydrogen adsorption‐free energies (Δ*G*_H*_) on the Fe_0.9_Co_0.1_S_2_(111), CoS_2_(111), FeS_2_(111), Fe_0.25_Co_0.75_S_2_(111), and α─Fe_2_O_3_(0001) surfaces are −0.06, 0.21, −0.45, 0.6, and −1.53 eV, respectively. The ΔG_H*_ value on the Fe_0.9_Co_0.1_S_2_(111) surface is the closest to the ideal value (Δ*G*_H*_ = 0), suggesting that it is also in the highest HER activity during the Heyrovsky/Tafel step. Overall, the theoretical predictions, aligning well with the experimental findings, demonstrate that blending Fe in the CoS_2_(111) surface, in particular Fe_0.9_Co_0.1_S_2_(111), exhibits enhanced catalytic activities for both OER and HER in the alkaline medium.

### 2.6 In‐Situ Probing Fe_x_Co_1‐x_S_2_‐Catalyzed OER and HER Processes

Since the Fe─OH─CoS_2_ showed the highest catalytic activities in alkaline OER and HER whose reaction thermodynamics was also confirmed by DFT calculations, it interested us to look into the real‐time processes taking place over the surfaces of CoS_2_─Fe_x_Co_1‐x_S_2_ nanooctahedra in the catalytic reactions. For this purpose, synchrotron X‐ray absorption (SXAS) and X‐ray emission spectroscopies (SXES) were taken for in‐situ measurements which were performed in 1 M KOH (pH = 13.8 ± 0.1) with an Ag/AgCl electrode as the reference electrode (RE), and a platinum wire as the counter electrode (CE). A specially designed Teflon container with a window sealed by Kapton tape was used as the electrolyzer.^[^ ^29^ ^]^ The Fe─OH─CoS_2_ was specifically investigated along with the CoS_2_ for comparison. Figure S20 (Supporting Information) collects the SXES spectra of Co/Fe Kβ_1, 3,_ and Kβ’ from the X‐ray absorption of Co/Fe K‐edges of CoS_2_ and Fe─OH─CoS_2_. In Figure S20a,b (Supporting Information), the Co Kβ' features for CoS₂ and Fe─OH─CoS_2_ underwent intensity reduction after immersing the sample‐loaded working electrodes into the KOH electrolyte at their corresponding open‐circuit potentials (OCP). It suggests significant reconfigurations in their Co electronic structures from the pristine to the pre‐catalyst states. More specifically, the weakened Co Kβ' signals reveal significant ligand‐to‐metal charge transfer upon interaction with the ‐OH ligands from OH^−^ anions in an alkaline medium, triggering the spin‐state conversion from higher to lower spin over the Co active sites. Additionally, the Co Kβ_1, 3_ signal of Fe─OH─CoS_2_ largely shifted to lower energy while that of CoS_2_ did very little. The changes represent the elevation in the Co oxidation states, and the larger shift reveals the higher activity of Co sites to OH^−^ anions which may lead to higher OER activity. Figure S20c (Supporting Information) is the SXES spectra of Fe Kβ_1, 3,_ and Kβ’ for Fe─OH─CoS_2_. The remaining strong Kβ’ features and unmoved Kβ_1,3_ signal infer the relative inertness of Fe sites to OH^−^ anions.

Given by the results that the catalysts would turn into the pre‐catalyst states at OCP in the KOH electrolyte, the catalyst‐loaded working electrodes (WE) were thus labeled as CoS₂_OCP and Fe─OH─CoS₂_OCP to be differentiated from their pristine states. In the in‐situ SXAS experiments, the CoS₂ and Fe─OH─CoS₂ WE were subjected to a potential of 1.5 V (vs RHE) to drive OER and labeled as CoS₂_1.5 V and Fe─OH─CoS₂_1.5 V, respectively. A reaction potential of −0.6 V (vs RHE) was applied for HER, and the WE labels were CoS₂_−0.6 V and Fe─OH─CoS₂_−0.6 V. Figure S21 (Supporting Information) collects the X‐ray absorption near‐edge structure (XANES) spectra of Co and Fe *K*‐edges for CoS₂ and Fe─OH─CoS₂ at different applied potentials. All spectra have weak pre‐edge features (arrows) arising from dipole‐allowed transitions, specifically from the 1s core level to the unoccupied 3d orbitals of metal elements.^[^ ^54^ ^]^ The weak features are influenced by the hybridization between metal 3d and ligand 2p orbitals (or neighbor‐coordinated atoms) within the crystal field. The shift in the pre‐edge intensity reflects the change in local crystal field symmetry, coordination number, metal‐ligand bond distance, and the degree of covalency in the metal‐ligand bond. Co and Fe *K*‐edges correspond to dipole‐allowed transitions from the 1s core level to unoccupied 4p‐derived orbitals. The intensity of near‐edge absorption peaks positively correlates with the number of unoccupied states in the orbitals. The shifts in the XANES spectra of CoS₂ and Fe─OH─CoS₂ under different potentials tell the evolutions in their electronic structures. Specifically, the potential‐dependent *K*‐edge energy shift to a higher value means oxidation and the lower value represents reduction. In Figure S21a (Supporting Information), the Co *K*‐edge for CoS₂ at OCP has an energy shift from 7714.9 to 7715.6 eV after elevating the potential to 1.5 V for driving OER while to 7714.8 eV after changing to −0.6 V for driving HER (Figure S21b, Supporting Information). On the other hand, the Co *K*‐edge for Fe─OH─CoS₂ has energy shifts from 7714.8 to 7716.6 and 7714.6 eV from OCP to 1.5 and −0.6 V, respectively (Figure S21c,d, Supporting Information). It's worth noting that the Co *K*‐edge of Fe─OH─CoS₂ exhibits a larger shift than that of CoS_2_ in the OER condition (ΔE_OER_: 0.7 vs 1.8 eV). In contrast, the energy‐shift difference is very little (ΔE_HER_: 0.1 vs 0.2 eV), and the morphologies of XANES profiles and the pre‐edge intensities for CoS₂ and Fe─OH─CoS₂ are also in little changes in the HER condition. The results infer that the activity enhancement for Co sites after blending Fe was much more significant for OER than for HER. Figure S21e (Supporting Information) shows the XANES spectra of Fe *K*‐edges for Fe─OH─CoS₂. The *K*‐edge energy shifts are from 7118.9 to 7124.0 and 7118.2 eV from OCP to 1.5 and −0.6 V (ΔE_OER_ = 5.1 eV and ΔE_HER_ = 0.7 eV), suggesting Fe is more sensitive to potential changes (Figure S21f, Supporting Information). However, the similar phenomenon in the edge shifts at the Co and Fe *K*‐edges of Fe─OH─CoS₂ fits with our estimation that the Fe─Co electronic effect exhibits more significant enhancement for OER over the Co sites.

**Figure 7:**
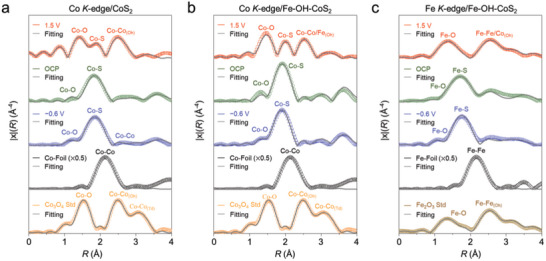
Optimized fitting results for χ(R) k^3^‐weighted FT EXAFS spectra of a) Co *K*‐edge of CoS_2_, b) Co *K*‐edge of Fe─OH─CoS_2_, c) Fe *K*‐edge of Fe─OH─CoS_2_ at different applied potentials, and the references of Co foil, Fe foil, Co_3_O_4_, and Fe_2_O_3_.

To have further insights into the results of XANES, the entended X‐ray absorption fine structure (EXAFS) spectra derived from Figure S21 (Supporting Information) by Fourier transform (FT) with k^3^‐weighting were conducted and analyzed. The purpose of k^3^‐weighting was chosen to improve the high‐k contributions in the FT data, allowing for a more detailed analysis of the coordination environment around Co or Fe sites. **Figure** **7** displays the k^3^‐weighted FT EXAFS spectra of the Co *K*‐edge of CoS_2_, and Co/Fe *K*‐edges of Fe─OH─CoS_2_ with optimized fitting at different applied potentials. The corresponding structural parameters derived from the fitting analyses are summarized in Tables S10 and S11 (Supporting Information). The magnitude of the FT spectra primarily reflects the coordination number (CN) and the scattering strength of neighboring atoms, which relate to the short‐range structural order. The radial distance represents the interatomic distance from the absorbing atom. At OCP, the Co‐S bond (2.30 Å) is observed as the major component (CN = 5.57) while the Co─O bond (1.87 Å) is minor (CN = 0.42) in the CoS₂ (Figure 7a). Similarly, the Co‐S (2.30 Å) bond with CN of 5.70 and the Cu─O bond (1.88 Å) with CN of 0.57 are determined in the Fe─OH─CoS_2_ (Figure 7b). The poor CN of Cu─O bond aligns with the observed Kβ_1, 3_ shifts of CoS_2_ and Fe─OH─CoS₂ in Figure S18a,b (Supporting Information), suggesting the evolved pre‐catalyst states on the nanooctahedral surfaces. The Fe *K*‐edge at OCP has (Figure 7c) the same phenomenon as the Co *K*‐edge. There are major Fe─S (2.24 Å, CN = 4.88) and minor Fe─O (1.88 Å, CN = 1.13) bonds observed.

At −0.6 V for HER, the CoS_2_ and Fe─OH─CoS₂ merely exhibited the Co/Fe─S bonds without detectable Co‐Co or Fe─Fe bonds. It reflects the durability of Co/Fe sites under the negative potentials as well as the effective prevention from the generation of Co/Fe─O oxides or hydroxides. However, the observed decreases in the average CN of Co‐S from 5.57 (OCP) to 5.14 (−0.6 V) in the CoS₂ and 5.70 (OCP) to 5.00 (−0.6 V) in the Fe─OH─CoS₂ reveal substantial sulfur leaching. The leaching also took place slightly over the Fe─S bonds from 4.88 (OCP) to 4.80 (−0.6 V) in the Fe─OH─CoS₂ (Table S10, Supporting Information). At 1.5 V for OER, the bonds of Co‐O (1.87–1.89 Å), Co‐Co/Fe (2.89 Å for Co at an octahedral site, and 3.45 Å for Co at a tetrahedral site), Fe─O (1.89 Å), and Fe─Fe/Co (3.28 Å) were generated in the CoS_2_ and Fe─OH─CoS₂ apart from the major Co‐S bonds (Table S10, Supporting Information). Those new‐born species correspond with the references of spinel Co_3_O_4_ and trigonal Fe_2_O_3_. The Co‐Co/Fe and Fe─Fe/Co bonds stand for the second‐shell coordination from the central Co or Fe cores. However, the Co‐Co, Co─Fe, and Fe─Fe are difficult to distinguish due to their close atomic sizes. The strong sulfur leaching happened over the Co and Fe sites in both CoS₂ and Fe─OH─CoS₂, where the CN numbers of Co decreased from 5.57 (OCP) to 1.12 (1.5 V) in the CoS₂ and from 5.70 (OCP) to 1.65 (1.5 V) in the Fe─OH─CoS₂. Notably, there is no residual Fe─S bond observed. All the EXAFS results suggest not only the drastic changes in the coordination environments of Co and Fe sites over the catalyst surfaces but also prove the involvement of OH^−^ in the OER mechanism that aligns with the results of the OER durability test in Figure S12 (Supporting Information).

## 3 Conclusion

By blending Fe into the CoS_2_ surfaces, the formed Fe_x_Co_1‐x_S_2_ faces with a variable Fe content exhibited higher activities in alkaline OER and HER, suggesting the enhancement by the Fe─Co electronic effect. Based on the comparison among the CoS_2_, Fe_0.25_Co_0.75_S_2_, and Fe_0.9_Co_0.1_S_2_ {111} surfaces on the nanooctahedra, the Fe_0.9_Co_0.1_S_2_ exhibited the most superior performance in both OER and HER with the low overpotentials η_100_ of 235 mV for OER and 342 mV for HER, and low Tafel slopes of 102 mV dec^−1^ for OER and 175 mV dec^−1^ for HER. Figures S22 and S23 summarize the comparison results among our work and literature for OER and HER, respectively. The overpotentials at 100 mA cm^−2^ (η_100_) and Tafel slopes of the Fe─OH─CoS_2_ in our work are comparable to those in the selected former reports, despite that only the overpotentials at 10 mA/cm^2^ (η_10_) are present in these literature. From the predictions by DFT calculations in reaction thermodynamics, the activities in OER and HER both follow the order of Fe_0.9_Co_0.1_S_2_(111) > Fe_0.25_Co_0.75_S_2_(111) > CoS_2_(111). From the validations by in‐situ SXAS and SXES in reaction kinetics, the Co sites of Fe_0.9_Co_0.1_S_2_(111) on the core‐shell nanooctahedra exhibited much higher activities than those of CoS_2_(111) under the applied potentials for OER and HER, which reflected the electronic benefits from the existing Fe neighbors. We believe that the direct evidence and systematic interpretations provide deep insights into the correlation between the Fe─Co electronic effect and catalyst activities for water splitting. More importantly, the results in this work will also offer a good model for future non‐noble‐metal catalyst designs for specific reactions.

## Conflict of Interest

The authors declare no conflict of interest.

## Supporting information



Supporting Information

## Data Availability

The data that support the findings of this study are available from the corresponding author upon reasonable request.
