# Supplementary material for: Turning the Surface Electronic Effect Over Core‐Shell CoS2─FexCo1‐xS2 Nanooctahedra Toward Electrochemical Water Splitting in the Alkaline Medium
Source: Adv Sci (Weinh). 2024 Nov 28;12(3):2411622. doi: 10.1002/advs.202411622 (PMC11744711; doi:10.1002/advs.202411622)
Supplement: Supplementary file 1 — Supporting Information [file ADVS-12-2411622-s001.docx]

Supporting Information

**Turning the Surface Electronic Effect over Core-Shell CoS_2_-Fe_x_Co_1-x_S_2_ Nanooctahedra towards Electrochemical Water Splitting in the Alkaline Medium**

Lian-Ming Lyu,^†^ Yu-Chung Chang,^†^ Han-Jung Li,^†^ Pei-En Wang, Ruei-Hung Juang, Ming-Yen Lu, Cheng-Shiuan Li, Chun-Hong Kuo^*^

**EXPERIMENTAL SECTION**

**Chemicals.** Cobalt(II) nitrate hexahydrate (Co(NO_3_)_2_∙6H_2_O, ≥ 98%, Sigma-Aldrich), iron (III) nitrate nonahydrate (Fe(NO_3_)_3_·9H_2_O 98.0-101.0% J. T. Baker), potassium hydroxide (KOH, ≥ 98%, Sigma-Aldrich), sodium thiosulfate 5-hydrate (Na_2_S_2_O_3_·5H_2_O 99.5%, J. T. Baker), carbon (paper, size 4 × 100 mm^2^/pc, 0.18 mm thick, CeTech), ethanol (EtOH, 95%, ECHO chemical), sulfuric acid (H_2_SO_4_, 95.0‒97.0%, Honeywell), Nafion-perfluorinated resin solution (5wt % in lower aliphatic alcohols and water, containing 15‒20% water, Sigma-Aldrich). In this study, all the chemicals were purchased from chemical vendors and used without purification. Ultrapure deionized water (DI water, 18.2 MΩ·cm) was used to prepare all solutions.

**Synthesis of** **Fe-OH**-**CoS_2,_ OH-CoS_2,_ Fe-CoS_2_, and CoS_2_ Nanocrystals.** The notations of Fe-OH-CoS_2_, OH-CoS_2_, and Fe-CoS_2_ represent the conditions when both of Fe^3+^ and OH^‒^ (Fe-OH-CoS_2_), only Fe^3+^ (OH-CoS_2_), and only Fe^3+^ were included for the syntheses, respectively. All the samples were synthesized using the stabilizer-free hydrothermal method. To synthesize Fe-OH-CoS_2_ nanoparticles, a 10 mL aqueous solution was prepared comprising 0.5 mmol of Co(NO_3_)_2_∙6H_2_O, 0.05 mmol of Fe(NO_3_)_3_∙9H_2_O, 0.5 mmol of KOH, and 1 mmol of Na_2_S_2_O_3_∙5H_2_O. The solution was vigorously stirred for 2 minutes before being transferred to a Teflon-lined stainless-steel autoclave with 20 mL in capacity. Subsequently, the mixture was heated at 180 ℃ in an air-circulating oven for 16 hours. Next, the solution was naturally cooled to room temperature. The resulting powder was collected by centrifugation at 5000 rpm and sequentially washed with deionized water, 25% ethanol, and 50% ethanol for bringing residual ions away. Finally, the powder was stored in 95% ethanol to keep the stability of the samples. Microspheres of OH-CoS_2_, Fe-CoS_2_, and CoS_2_ were obtained using the same procedure except that Fe(NO_3_)_3_∙9H_2_O was excluded for OH-CoS_2_, KOH was omitted for Fe-CoS_2_, and only Co(NO_3_)_2_∙6H_2_O and Na_2_S_2_O_3_∙5H_2_O were included for CoS_2_.

**Characterization.** Morphologies and surface states of nanocrystals were analyzed by field-emission scanning electron microscopy operated at an accelerated voltage of 10 keV (FE-SEM, JEOL JSM6700F). Bright-field TEM and HAADF-STEM-EDS images were acquired by spherical-aberration-corrected field-emission transmission electron microscopy (Cs-corrected TEM, JEOL ARM 200 F) operated at 200 keV. The crystal structures of the samples were identified using XRD (Bruker D8 Advance diffractometer) with Cu Kα radiation. The elemental ratios of all samples were measured by the inductively coupled plasma with mass spectroscopy (NexION 350 ICP-MS, PerkinElmer). The electronic states of elements for all samples were determined by a high-resolution X-ray photoelectron spectrometer (ULVAC-PHI, PHI Quantera II) equipped with a scanning X-ray microprobe (Al anode) as the X-ray source. Powder X-ray diffraction (PXRD) patterns were collected at TPS 19A in NSRRC, Taiwan by using 20 keV hard X-ray source (0.61992 Å in wavelength). The patterns were converted by the GSAS-II program. LaB_6_ (SRM 660c) standard was used for angle calibration. Operando absorption experiments were carried out using Taiwan Photon Source in the National Synchrotron Radiation Research Center (NSRRC), Taiwan. The XAS spectra including X-ray absorption near-edge structure (XANES) and extended X-ray absorption fine structure (EXAFS), were obtained at TPS44A with the q-scan method in the transmission mode. Standard metal foils (Fe and Co) were employed as references for the energy calibration of incident photons. Each spectrum was collected with Q-Mono oscillating at 1 Hz for 2 minutes. Data fitting of XANES and EXAFS were conducted with Demeter package.

**Electrochemical Hydrogen and Oxygen Evolution Reactions.** Chosen as the substrate of the working electrode, the carbon paper was fixed by PTFE platinum electrode holder. 20 mg of electrocatalysts were dispersed in a mixed solution (180 μL of ethanol, and 20 μL of 5 wt% Nafion solution). The solution was sonicated for at least 10 minutes to form homogeneous ink. 10 μL of the ink was loaded onto the carbon paper electrode. The amount of loading was about 3 ± 0.3 mg/cm^2^_._

All the measurements of oxygen evolution reaction (OER) were carried out on a three-electrode potentiostat (CHI 760E, CH Instruments) at room temperature (25 ℃). A cylinder-shaped plastic cell was utilized to hold the electrolyte and set the electrodes. Electrochemical experiments were conducted in the electrolyte of 1 M KOH (pH = 13.8 ± 0.1). A graphite rod was used as the counter electrode, and a Hg/HgO was used as the reference electrode. The geometric area of the working electrode immersed in the electrolyte was 0.3 × 1 cm^2^ used for datum normalization. The detailed investigations and linear sweep voltammogram (LSV) were done across the range from −0.9 to −1.7 V (vs Hg/HgO) for HER, and from 0.1 to 1 V (vs Hg/HgO) for OER at a scan rate of 50 mV/s. For OER LSV measurements, 85% iR-correction was carried out while those of HER were not. The OER LSV curves without 85% iR-correction are shown in Figure S8 for reference. All the applied potentials were ultimately converted to the values versus reversible hydrogen electrode (RHE) by the following equation:

*E*_RHE_ = *E*_Hg/HgO_ + 0.118 + 0.0591 × pH (1)

The electrochemical double-layer capacitance (C_dl_) was evaluated by collecting cyclic voltammogram (CVs) in the potential range with a nonfaradic process at different scan rates (*r*) of 10, 20, 40, 60, 80, and 100 mV/s. The potential windows of ECSA were determined by the open-circuit potential (OCP) ± 0.04 V (vs RHE). Then, the double-layer capacitance (C_dl_), which is half of d(Δ*I*)/d(*v*), was estimated by plotting Δ*I*_OCP_ = (*I*_top_ − *I*_bottom_ at OCP) as a function of the scan rate (*v*). The electrochemical active surface areas (ECSAs) were estimated according to the following formula.

ECSA = C_dl_/C_s_ (2)

C_s_ is the specific capacitance of a flat surface with 1 cm^2^ of real surface area, which is generally in the range of 0.02 to 0.06 mF/cm^2^. Thus, the averaged value of 0.04 mF/cm^2^ was assumed for the flat electrode. Electrochemical impedance spectroscopy (EIS) was conducted by applying the potentials of 1.45 and −0.45 V (vs RHE) for OER and HER, AC frequency from 100 kHz to 0.1 Hz, and an amplitude of 0.005 V. In the chronopotentiometry (CP) test, the time-dependent variation in the potential at a static current density of 100 mA cm^-2^ was examined for 100 hours.

**Density Functional Theory Calculations.** All calculations were performed using the DFT plane-wave method as implemented in the Vienna Ab initio Simulation Package (VASP). The projector-augmented-wave (PAW) pseudopotentials with a cutoff energy of 400 eV, in conjunction with the Perdew-Burke-Ernzerhof (PBE) density functional, were employed.^1-4^ The on-site Coulomb potential (DFT+U) correction with a U value of 4.0 eV was applied to the 3d electrons of Fe atoms in the α-Fe_2_O_3_(0001) surface.^5, 6^ The CoS_2_(111), Fe_0.25_Co_0.75_S_2_(111), and FeS_2_(111) surfaces were modeled with four layers of metal atoms, while the Fe_0.9_Co_0.1_S_2_(111) surface was modeled with five layers. The α-Fe_2_O_3_(0001) surface was simulated with six layers of metal atoms. All surfaces were modeled using a (2 × 2) lateral supercell. During the structural optimization calculations, half of the atomic layers were fixed. To mitigate any undesirable interactions along the z-direction, a vacuum of at least 15 Å was introduced. For the summation in the Brillouin zone, the Monkhorst−Pack mesh k-point was set to (4 × 4× 1) at all surfaces.^7^ The oxygen evolution reaction (OER) was modeled at the active sites (*) based on the four-electron mechanism proposed by Nørskov.^8, 9^ The reaction steps are as follows:

OH^−^ + * → OH* + e^−^ ∆G1 (3)

OH* → O* + H^+^ + e^−^ ∆G2 (4)

O* + OH^−^ → OOH* + e^−^ ∆G3 (5)

OOH^*^ → O_2_ + * + H^+^ + e^−^ ∆G4 (6)

where the *, OH*, O*, and OOH^*^ represent the pure surface, adsorbed OH, O, and OOH species, respectively. For hydrogen evolution reaction (HER), theoretical work by Nørskov et al. demonstrated that as inferred from the kinetic model, the HER activity strongly correlates with the free energy of H adsorption (Δ*G*_H*_) onto the electrocatalyst surface.^10-12^ Accordingly, Δ*G*_H*_ has been widely employed as a descriptor for the HER activity. The adsorption energy of H (Δ*E*_H_*_*_*) was calculated based on the equation as follows:

Δ*E*_H*_ = *E*(surface + H) − [ *E*(surface) + ½ *E*(H_2_) ] (7)

Then, the free energy of H adsorption, Δ*G*_H*_, was calculated according to the following equation:

Δ*G*_H*_ = Δ*E*_H*_ + Δ*E*_ZPE_ − *T*Δ*S*_H_ (8)

where Δ*E*_H*_, Δ*E*_ZPE_, and Δ*S*_H_ represent the changes of electronic energy, zero-point energy, and entropy from a gas phase to an adsorbed hydrogen, respectively. The climbing-image nudged-elastic-band (CI-NEB) method was used to locate the transition structures and frequency calculations were used to verify the transition states.^13^

**In-Situ Synchrotron X-Ray Absorption and Emission Spectroscopy.** The measurements were conducted using quick-scanning X-ray absorption spectroscopy (XAS) at the TPS BL44A station in the National Synchrotron Radiation Research Center (NSRRC) in Hsinchu City, Taiwan. Specifically, the XAS spectra for the Fe and Co *K*-edges were acquired in transmission mode, with Fe and Co metal foils serving as energy calibration references. The spectral energy resolution was set at 0.3 eV for 7112 and 7709 eV photon energies, corresponding to the Fe and Co *K*-edges, respectively. XAS was utilized to determine the local structure around the absorbing atoms in the catalysts, including the coordination shell, structural disorder, and interatomic distances. The X-ray absorption near-edge structure (XANES) provides insights into the electronic structure and local geometric information. In contrast, the extended X-ray absorption fine structure (EXAFS) yields detailed information on the local atomic structure around the absorbing atoms, such as the type of nearest neighboring atoms and their specific numbers.

All XAS data were analyzed using the Demeter software package, including Athena for data normalization and Artemis for best-fit fitting. The fitting was performed over the k-range of 3 to 11.5 Å⁻¹ and the R-range of 1 to 3.5 Å for all samples. Here, N represents the coordination number, R is the distance between the absorber and backscatter atoms, σ² is the Debye-Waller factor, and Rf is the R-factor, indicating the goodness of fit. The amplitude reduction factor (S₀²) was fixed at 0.7, as determined from Fe and Co foils. The error bounds (accuracies) for the structural parameters obtained from the EXAFS data analysis are estimated to be N, ± 20%; R, ± 1%; and σ², ± 20%. X-ray emission spectroscopy (XES) was performed at the Taiwan beamline 12XU in Spring-8, Japan. The incident beam energy, which was constant at 7142 eV for Fe Kβ and 7739 eV for Co Kβ, was monochromatized by a Si (111) double-crystal monochromator. The monochromatized beam was allowed to transmit through the tape and electrolyte at a 0° incident angle. The fluorescence was split by the analyzer crystal Si (444), with signals collected by a silicon drift detector (XR-100CR Si-PIN X-ray detector) in the scan range of 7030–7075 eV for Fe K_β_ and 7720–7770 eV for Co K_β_ in BL-12XU at Spring-8.

**Statistical Analysis.** The size-distribution histograms of particle sizes were conducted by using ImageJ (ver. 1.54g) to measure at least 100 particles and followed by calculations with Microsoft Excel (2019) for getting the values of average size and standard deviations. The standard deviations for overpotentials were also calculated by Microsoft Excel (2019).

**Figure *S1*.** Size-distribution histograms of (a) CoS_2_, (b) OH-CoS_2_, (c) Fe-CoS_2_, and (d) Fe-OH-CoS_2_.

***Figure S2.*** Rietveld refinement for SPXRD patterns of (a) CoS_2_, (b) OH-CoS_2_, (c) Fe-CoS_2_, and (d) Fe-OH-CoS_2_.

***Table S1.*** Quantitative ratios of crystal components in all synthesized nanocrystals.

| Label | Phase | Phase Ratio | Weight Ratio | Mole Ratio |
| --- | --- | --- | --- | --- |
| CoS_2_ | CoS_2_ | 1.00 | 1.00 | 1.00 |
| OH-CoS_2_ | CoS_2_ | 1.00 | 1.00 | 1.00 |
| Fe-CoS_2_ | CoS_2_ | 0.6325 | 0.622 | 0.627 |
|  | Fe_0.248_Co_0.752_S_2_ | 0.3429 | 0.331 | 0.337 |
|  | Fe_2_O_3_ | 0.0245 | 0.047 | 0.036 |
| Fe-OH-CoS_2_ | CoS_2_ | 0.7885 | 0.792 | 0.788 |
|  | Fe_0.904_Co_0.096_S_2_ | 0.2115 | 0.208 | 0.212 |

***Table S2*.** Crystallographic data of CoS_2_ nanocrystals.

| Formula | CoS_2_ |
| --- | --- |
| Molecular weight (g/mol) | 123.05 |
| Wavelength (Å) | 0.61992 |
| Space group | P a -3 |
| 2θ range (°) | 9.5 to 90.994 |
| a (Å) | 5.52709(5) |
| b (Å) | 5.52709 |
| c (Å) | 5.52709 |
| Volume (Å^3^) | 168.845(5) |
| Size (μm) | 0.514 |
| Microstrain | 21521.7 |
| wRp | 6.92% |
| Zero point shift (°) | 0.0004 |
| Uiso S | 0.00066 |
| Uiso Co | 0.00039 |
| RF^2^ | 4.92% |

***Table S3*.** Crystallographic data of OH-CoS_2_ nanocrystals.

| Formula | CoS_2_ |
| --- | --- |
| Molecular weight (g/mol) | 123.05 |
| Wavelength (Å) | 0.61992 |
| Space group | P a -3 |
| 2θ range (°) | 2.002 to 90.994 |
| a (Å) | 5.531609(21) |
| b (Å) | 5.531609 |
| c (Å) | 5.531609 |
| Volume (Å^3^) | 169.2600(19) |
| Size (μm) | 0.218 |
| Microstrain | 12138.2 |
| wRp | 4.84% |
| Zero point shift (°) | 0.0004 |
| Uiso S | 0.00109 |
| Uiso Co | 0.00205 |
| RF^2^ | 3.36% |

***Table S4*.** Crystallographic data of Fe-CoS_2_ nanocrystals.

| Formula | CoS_2_ | Fe_0.248_Co_0.752_S_2_ | Fe_2_O_3_ |
| --- | --- | --- | --- |
| Molecular weight (g/mol) | 123.05 | 120.72 | 159.70 |
| Wavelength (Å) | 0.61992 | | |
| Space group | P a -3 | P a -3 | R -3 c |
| 2θ range (°) | 10.75 to 90.994 | | |
| a (Å) | 5.520905(28) | 5.50373(6) | 5.03595(18) |
| b (Å) | 5.520905 | 5.50373 | 5.03595 |
| c (Å) | 5.520905 | 5.50373 | 13.7906(4) |
| Volume (Å^3^) | 168.2794(26) | 166.714(5) | 302.885(11) |
| Size (μm) | 0.594 | 0.300 | 0.078 |
| Microstrain | 12883.0 | 12022.9 | 1030.0 |
| wRp | 5.07% | | |
| Zero point shift (°) | 0.0004 | | |
| Uiso S | 0.00344 | 0.0734 | 0.02279 |
| Uiso Co | 0.00131 | 0.0645 | 0.03136 |
| Uiso Fe | - | 0.0389 | 0.02634 |
| Uiso O | - | - | 0.01693 |
| RF^2^ | 3.12% | 3.00% | 17.36% |

***Table S5*.** Crystallographic data of Fe-OH-CoS_2_ nanocrystals.

| Formula | CoS_2_ | Fe_0.904_Co_0.096_S_2_ |
| --- | --- | --- |
| Molecular weight (g/mol) | 123.05 | 120.26 |
| Wavelength (Å) | 0.61992 | |
| Space group | P a -3 | P a -3 |
| 2θ range (°) | 10.0 to 90.994 | |
| a (Å) | 5.51574(6) | 5.45342(22) |
| b (Å) | 5.51574 | 5.45342 |
| c (Å) | 5.51574 | 5.45342 |
| Volume (Å^3^) | 167.808(6) | 162.183(20) |
| Size (μm) | 0.975 | 0.302 |
| Microstrain | 26352.8 | 22569.2 |
| wRp | 6.38% | |
| Zero point shift (°) | 0.0004 | |
| RF^2^ | 6.79% | 6.49% |
| Uiso S | 0.01378 | 0.00787 |
| Uiso Co | 0.00175 | 0.01666 |
| Uiso Fe | - | 0.01330 |
| RF^2^ | 6.79% | 6.49% |

***Table S6*.** The ICP-MS results for the Fe-OH-CoS_2_.

| **Fe-OH-CoS_2_** | **Co/ppm** | **Fe/ppm** | **Fe%** |
| --- | --- | --- | --- |
| value | 442.46 | 39.26 | 8.55 ± 0.08 |

***Figure S3.*** The HAADF-STEM images and their STEM-EDX elemental maps of (a-c) CoS_2_, (d-f) OH-CoS_2_, and (g-j) Fe-CoS_2_.

***Figure S4.*** (a) The PXRD pattern and (b) SEM image of Fe-OH synthesized without addition of Co(NO_3_)_2_. (c) The BF-TEM image of a single nanoplate from the Fe-OH sample, and (d) its corresponding SAED pattern acquired along the [0001] zone axis.

***Figure S5.*** Deconvoluted HRXPS patterns of (a-d) Co(2p), and (e-f) Fe(2p) for CoS_2_, OH-CoS_2_, Fe-CoS_2_, and Fe-OH-CoS_2_.

***Figure S6.*** Deconvoluted HRXPS spectra of (a-d) O(1s) and (e-h) S(2p) for CoS_2_, OH-CoS_2_, Fe-CoS_2_, Fe-OH-CoS_2_.

***Figure S7.*** (a-e) CVs obtained by scanning at the OCP at different scan rates (*r*) of 10, 20, 40, 60, 80, and 100 mV/s and (f) their corresponding plots of Δ*I*_OCP_/2 vs *r* for (a) CoS_2_ (b) OH-CoS_2_, (c) Fe-CoS_2_, (d) Fe-OH-CoS_2_, and (e) Fe_2_O_3_.

***Figure S8.*** The OER LSV curves of different catalysts obtained at 50 mV/s without 85% iR-correction.

***Figure S9.*** The Nyquist plots obtained by applying the potentials of (a) 1.45 V for OER and (b) -0.45 V for HER (vs RHE).

***Table S7.*** EIS fitting results of different Co-based pyrite electrocatalysts for OER.

|  | R_s_ (ꭥ) | R_f_ (ꭥ) | R_ct_ (ꭥ) | CPE_1T_  (mFs^a-1^) | CPE_1P_  (= a) | CPE_2T_  (mFs^a-1^) | CPE_2P_  (= a) |
| --- | --- | --- | --- | --- | --- | --- | --- |
| CoS_2_ | 8.119 | 5.07 | 12.06 | 0.0084 | 0.6710 | 0.0002 | 0.7792 |
| Fe-CoS_2_ | 6.624 | 1.82 | 7.70 | 0.0146 | 0.7048 | 0.0026 | 0.6274 |
| OH-CoS_2_ | 7.395 | 3.838 | 9.34 | 0.0116 | 0.7002 | 0.0011 | 0.5949 |
| Fe-OH-CoS_2_ | 6.361 | 1.39 | 5.05 | 0.0210 | 0.7587 | 0.0038 | 0.5523 |

***Table S8.*** EIS fitting results of different Co-based pyrite electrocatalysts for HER.

|  | R_s_ (ꭥ) | R_ct_ (ꭥ) | CPE_1T_  (mFs^a-1^) | CPE_1P_  (= a) |
| --- | --- | --- | --- | --- |
| CoS_2_ | 6.82 | 28.77 | 0.004 | 0.868 |
| Fe-CoS_2_ | 4.80 | 18.77 | 0.006 | 0.911 |
| OH-CoS_2_ | 5.50 | 14.82 | 0.006 | 0.886 |
| Fe-OH-CoS_2_ | 5.60 | 14.00 | 0.004 | 0.869 |

***Figure S10.*** The results of electrocatalytic (a-c) OER and (d-f) HER measured in 1 M KOH electrolyte. (a, d) Polarization curves for 0.5Fe-OH-CoS_2_, 1.0Fe-OH-CoS_2_, and 1.5Fe-OH-CoS_2_ at 50 mV/s. (b, e) Overpotentials required for current density at 100 mA/cm^2^ (OER), and −100 mA/cm^2^ (HER). (c, f) Tafel plots obtained from the polarization curves.

***Table S9.*** Atomic ratios of Fe, Co, and S in the pyrite samples with different Fe amounts.

|  | Fe (%) | Co (%) | S (%) |
| --- | --- | --- | --- |
| 0.5Fe-OH-CoS_2_ | 3.6 | 26.0 | 70.4 |
| 1.0Fe-OH-CoS_2_ | 4.0 | 26.3 | 69.7 |
| 1.5Fe-OH-CoS_2_ | 9.8 | 25.4 | 64.7 |

***Figure S11.*** HAADF-STEM-EDX maps of (a-d) 0.5Fe-OH-CoS_2_, (e-h) 1.0Fe-OH-CoS_2_, and (i-l) 1.5Fe-OH-CoS_2_.

***Figure S12.*** The results of chronopotentiometry (CP) testing on the Fe-OH-CoS_2_ electrocatalyst in OER and HER for 100 hours. The arrows indicate the timing of renewing the electrolyte of KOH.

***Figure S13.*** HAADF-STEM images and the STEM-EDX maps of Co, Fe, S, and O for the Fe-OH-CoS_2_ after (a-e) OER and (f-j) HER for 100 hours.

***Figure S14.*** Deconvoluted HRXPS patterns of Co(2p), Fe(2p), S(2p), and O(1S) for the Fe-OH-CoS_2_ after (a-d) OER and (e-h) HER for 100 hours

***Figure S15.*** The results of chronoamperometry (CA) testing on the CoS2, Fe-CoS2, and OH-CoS2 in (a) OER and (b) HER for 17 hours.

***Figure S16.*** Top and side views of various surfaces. The blue, yellow, brown, and red spheres represent Co, S, Fe and O atoms, respectively.

***Figure S17.*** Optimized *OH, * O, and * OOH OER intermediates on various surfaces. The blue, yellow, brown, red and pink spheres represent Co, S, Fe, O and H atoms, respectively.

***Figure S18.*** Optimized structures of initial, transition, and final states for breaking the H-OH bond in the Volmer step on various surfaces. The blue, yellow, brown, red and pink spheres represent Co, S, Fe, O and H atoms, respectively.

***Figure S19.*** Optimized structures of H adsorption on various surfaces. The blue, yellow, brown, red and pink spheres represent Co, S, Fe, O and H atoms, respectively.

***Figure S20.*** XES spectra of (a, b) Co Kβ_1, 3_ and Kβ’ from (a) CoS_2_ and (b) Fe-OH-CoS_2_, and (c) Fe Kβ_1, 3_ and Kβ’ from Fe-OH-CoS_2_.

***Figure S21.*** XANES spectra of CoS_2_ and Fe-OH-CoS_2_. (a, b) Co *K*-edge spectra of CoS_2_. (c, d) Co *K*-edge spectra of Fe-OH-CoS_2_. (e, f) Fe *K*-edge spectra of Fe-OH-CoS_2_.

***Table S10*.** Structural parameters obtained from Co and Fe *K*-edge EXAFS fitting analysis for the samples used in HER conditions.

|  | Scattering Path | N | σ^2^(Å^2^) | R(Å) | R_f_ |
| --- | --- | --- | --- | --- | --- |
| Co Foil | Co-Co | 12 | 0.005 | 2.48 | 0.0005 |
| CoS_2_­_OCP | Co-O | 0.42 | 0.004 | 1.87 | 0.01 |
|  | Co-S | 5.57 | 0.004 | 2.30 |  |
| CoS_2_­_−0.6 V | Co-O | 0.34 | 0.006 | 1.87 | 0.01 |
|  | Co-S | 5.14 | 0.006 | 2.89 |  |
| Fe-OH-CoS_2_­_OCP | Co-O | 0.57 | 0.005 | 1.88 | 0.01 |
|  | Co-S | 5.7 | 0.005 | 2.30 |  |
| Fe-OH-CoS_2_­_−0.6 V | Co-O | 0.42 | 0.004 | 1.88 | 0.013 |
|  | Co-S | 5.0 | 0.004 | 2.29 |  |

|  | Scattering Path | N | σ^2^(Å^2^) | R(Å) | R_f_ |
| --- | --- | --- | --- | --- | --- |
| Fe foil | Fe-Fe | 8 | 0.004 | 2.47 | 0.01 |
| Fe-OH-CoS_2_­_OCP | Fe-O | 1.13 | 0.004 | 1.88 | 0.02 |
|  | Fe-S | 4.88 | 0.004 | 2.24 |  |
| Fe-OH-CoS_2_­_−0.6 V | Fe-O | 1.11 | 0.003 | 1.90 | 0.02 |
|  | Fe-S | 4.8 | 0.003 | 2.25 |  |

Fitting was done across the *k* range of 3 to 11.5 Å^–1^ and the *R* range of 1 to 3.5 Å for all samples. Where N is the coordination number, R is the distance between the absorber and backscatter atoms, σ^2^ is the Debye-Waller factor, and R_f_ is the R-factor characterizing the goodness of fitting. S_o_^2^ was fixed to 0.7, as determined by Co and Fe foil. Error bounds (accuracies) characterizing the structural parameters obtained by EXAFS data analysis are estimated to be as follows: N, ±20%; R, ±1%; and σ^2^, ±20%.

***Table S11.*** Structural parameters obtained from Co and Fe *K*-edge EXAFS fitting analysis for the samples used in OER conditions.

|  | Scattering Path | N | σ^2^(Å^2^) | R(Å) | R_f_ |
| --- | --- | --- | --- | --- | --- |
| Co Foil | Co-Co | 12 | 0.005 | 2.48 | 0.0005 |
| Co_3_O_4_-Std | Co-O | 4 | 0.006 | 1.90 | 0.004 |
|  | Co-Co_(Oh)_ | 12 | 0.006 | 2.86 |  |
|  | Co-Co_(Td)_ | 12 | 0.006 | 3.37 |  |
| CoS_2_­_OCP | Co-O | 0.42 | 0.004 | 1.87 | 0.01 |
|  | Co-S | 5.57 | 0.004 | 2.30 |  |
| CoS_2_­_1.5 V | Co-O | 4.35 | 0.01 | 1.89 | 0.002 |
|  | Co-S | 1.12 | 0.01 | 2.34 |  |
|  | Co-Co_(Oh)_ | 2.23 | 0.005 | 2.89 |  |
|  | Co-Co_(Td)_ | 1.26 | 0.005 | 3.45 |  |
| Fe-OH-CoS_2_­_OCP | Co-O | 0.57 | 0.005 | 1.88 | 0.02 |
|  | Co-S | 5.7 | 0.005 | 2.30 |  |
| Fe-OH-CoS_2_­_1.5 V | Co-O | 3.49 | 0.003 | 1.89 | 0.004 |
|  | Co-S | 1.65 | 0.003 | 2.31 |  |
|  | Co-Co_(Oh)_ | 2.44 | 0.005 | 2.89 |  |
|  | Co-Co_(Td)_ | 1.91 | 0.005 | 3.4 |  |

|  | Scattering Path | N | σ^2^(Å^2^) | R(Å) | R_f_ |
| --- | --- | --- | --- | --- | --- |
| Fe foil | Fe-Fe | 8 | 0.004 | 2.47 | 0.01 |
| Fe_2_O_3_-Std | Fe-O | 4 | 0.008 | 1.9 | 0.01 |
|  | Fe-Fe_(Oh)_ | 12 | 0.003 | 3.28 | 0.01 |
| Fe-OH-CoS_2_­_OCP | Fe-O | 1.13 | 0.004 | 1.88 | 0.02 |
|  | Fe-S | 4.88 | 0.004 | 2.24 |  |
| Fe-OH-CoS_2_­_1.5 V | Fe-O | 3.9 | 0.003 | 1.89 | 0.01 |
|  | Fe-Fe_(Oh)_ | 9.37 | 0.003 | 3.28 | 0.01 |

Fitting was done across the *k* range of 3 to 11.5 Å^–1^ and the *R* range of 1 to 3.5 Å for all samples. Where N is the coordination number, R is the distance between the absorber and backscatter atoms, σ^2^ is the Debye-Waller factor, and R_f_ is the R-factor characterizing the goodness of fitting. S_o_^2^ was fixed to 0.7, as determined by Co and Fe foil. Error bounds (accuracies) characterizing the structural parameters obtained by EXAFS data analysis are estimated to be as follows: N, ±20%; R, ±1%; and σ^2^, ±20%.

***Figure S22.*** Comparison of overpotentials and Tafel slopes in alkaline OER among this work and selected literatures.

***Figure S23.*** Comparison of overpotentials and Tafel slopes in alkaline HER among this work and selected literatures.

**REFERENCES**

[1] G. Kresse, J. Furthmuller, *Phys. Rev. B* **1996**, *54*, 11169.

[2] P. E. Blöchl, *Phys. Rev. B* **1994**, *50*, 17953.

[3] G. Kresse, D. Joubert, *Phys. Rev. B* **1999**, *59*, 1758.

[4] J. P. Perdew, K. Burke, M. Ernzerhof, *Phys. Rev. Lett.* **1996**, *77*, 3865.

[5] I. Ahamed, K. Ulman, N. Seriani, R. Gebauer, A. Kashyap, *J. Chem. Phys.* **2018**, *148*, 214707.

[6] N. Kumar, N. Seriani, R. Gebauer, *Phys. Chem. Chem. Phys.* **2020**, *22*, 10819.

[7] H. J. Monkhorst, J. D. Pack, *Phys. Rev. B* **1976**, *13*, 5188.

[8] J. K. Norskov, J. Rossmeisl, A. Logadottir, L. Lindqvist, J. R. Kitchin, T. Bligaard, H. Jónsson, *J. Phys. Chem. B* **2004**, *108*, 17886.

[9] J. Rossmeisl, Z. W. Qu, H. Zhu, G. J. Kroes, J. K. Norskov, *J. Electroanal. Chem.* **2007**, *607*, 83. [10] J. K. Norskov, T. Bligaard, A. Logadottir, J. R. Kitchin, J. G. Chen, S. Pandelov, J. K. Norskov, *J. Electrochem. Soc.* **2005**, *152*, J23.

[11] J. Greeley, J. K. Norskov, L. A. Kibler, A. M. El-Aziz, D. M. Kolb, *ChemPhysChem* **2006**, *7*, 1032.

[12] J. Greeley, T. F. Jaramillo, J. Bonde, I. B. Chorkendorff, J. K. Norskov, *Nat. Mater.* **2006**, *5*, 909.

[13] G. Henkelman, B. P. Uberuaga, H. Jónsson, *J. Chem. Phys.* **2000**, *113*, 9901.

[14] S. B. Devi, R. Navamathavan, *J. Electrochem. Soc.* **2023**, *170*, 096503.

[15] J. Nan, B. Ye, S. Peng, W. Zhang, H. Liu, Y. Zhang, *Mater.s Lett.* **2024**, *354*, 135323.

[16] B. Fang, Y. Li, J. Yang, T. Lu, X. Liu, X. Chen, L. Pan, Z. Zhao, *ACS Appl. Nano Mater.* **2024**, *7*, 9685.

[17] L. Zhu, L. Liu, G. Huang, Q. Zhao, *Appl. Surf. Sci.* **2020**, *504*, 144490.

[18] H. Khan, S. A. Shah, W. U. Rehman, F. Chen, *Adv. Mater. Interfaces* **2022**, *9*, 2101294.

[19] Y. Li, Q.-X. Du, J. Cui, H.-W. Yang, H. Qian, *Inorg. Chem.* **2024**, *63*, 1954.

[20] U. Aftab, A. Tahira, A. H. Samo, M. I. Abro, M. M. Baloch, M. Kumar, Sirajuddin, Z. H. Ibupoto, *Int. J. Hydrogen Energy* **2020**, *45*, 13805.

[21] A. Mariappan, R. K. Dharman, T. H. Oh, *Ceram. Int.* **2023**, *49*, 29984.

[22] K. Karuppasamy, R. Bose, V. R. Jothi, D. Vikraman, Y.-T. Jeong, P. Arunkumar, D. B. Velusamy, T. Maiyalagan, A. Alfantazi, H.-S. Kim, *J. Alloys Compd.* **2020**, *838*, 155537.

[23] N. Li, S. Qu, J. Ma, W. Shen, *Int. J. Hydrogen Energy* **2023**, *48*, 180.

[24] X. Hu, P. Tan, R. Dong, M. Jiang, L. Lu, Y. Wang, H. Liu, Y. Liu, J. Xie, J. Pan, *Energy Technol.* **2021**, *9*, 2000961.

[25] L. Zhao, C. Gong, X. Chen, X. He, H. Chen, X. Du, D. Wang, W. Fang, H. Zhang, W. Li, *Appl. Surf. Sci.* **2023**, *623*, 157030.

[26] C. Wang, H. Xu, Y. Wang, H. Shang, L. Jin, F. Ren, T. Song, J. Guo, Y. Du, *Inorg. Chem.* **2020**, *59*, 11814.

[27] W. Peng, A. Deshmukh, N. Chen, Z. Lv, S. Zhao, J. Li, B. Yan, X. Gao, L. Shang, Y. Gong, L. Wu, M. Chen, T. Chang, H. Gou, *ACS Catal.* **2022**, *12*, 3743.

[28] A. Mondal, H. R. Inta, A. Roy, A. Kumar Mahato, V. Mahalingam, *ACS Appl. Nano Mater.* **2023**, *6*, 12040.

[29] J. Yu, Y. Qian, S. Seo, Y. Liu, H. T. D. Bui, N. Q. Tran, J. Lee, A. Kumar, H. Wang, Y. Luo, X. Shao, Y. Cho, X. Liu, M. G. Kim, H. Lee, *J. Energy Chem.* **2023**, *85*, 11.

[30] C. Song, J. Yang, C. Ayappan, S. K. Kannan, H. Yang, R. Xing, H. Yang, S. Liu, *Colloids Surf. A: Physicochem. Eng.* **2024**, *692*, 133962.
